# Randomized controlled trial on the influence of dietary intervention on epigenetic mechanisms in children with cow’s milk allergy: the EPICMA study

**DOI:** 10.1038/s41598-019-38738-w

**Published:** 2019-02-26

**Authors:** Lorella Paparo, Rita Nocerino, Cristina Bruno, Carmen Di Scala, Linda Cosenza, Giorgio Bedogni, Margherita Di Costanzo, Maurizio Mennini, Valeria D’Argenio, Francesco Salvatore, Roberto Berni Canani

**Affiliations:** 1 Department of Translational Medical Science, University Federico II, Naples, Italy; 2 Clinical Epidemiology Unit, Liver Research Center, Basovizza, Trieste, Italy; 3 Hospital Bambino Gesù in Rome, Vatican City, Italy; 4 CEINGE-Biotecnologie Avanzate s.c.ar.l., University Federico II, Naples, Italy; 5 Department of Molecular Medicine and Medical Biotechnologies, University Federico II, Naples, Italy; 6 European Laboratory for the Investigation of Food-Induced Diseases, University Federico II, Naples, Italy; 7 Task Force on Microbiome Studies, University Federico II, Naples, Italy

**Keywords:** Gene regulation, Epigenetics in immune cells

## Abstract

Epigenetic mechanisms could drive the disease course of cow’s milk allergy (CMA) and formula choice could modulate these pathways. We compared the effect of two different dietary approaches on epigenetic mechanisms in CMA children. Randomized controlled trial on IgE-mediated CMA children receiving a 12-month treatment with extensively hydrolyzed casein formula containing the probiotic *L.rhamnosus* GG (EHCF + LGG) or with soy formula (SF). At the baseline, after 6 and 12 months of treatment *FoxP3* methylation rate and its expression in CD4^+^ T cells were assessed. At same study points IL-4, IL-5, IL-10, and IFN-γ methylation rate, expression and serum concentration, miRNAs expression were also investigated. 20 children (10/group) were evaluated. Baseline demographic, clinical and epigenetic features were similar in the two study groups. At 6 and 12 months, EHCF + LGG group showed a significant increase in *FoxP3* demethylation rate compared to SF group. At the same study points, EHCF + LGG group presented a higher increase in IL-4 and IL-5 and a higher reduction in IL-10 and IFN-γ DNA methylation rate compared to SF group. A different modulation of miR-155, -146a, -128 and -193a expression was observed in EHCF + LGG vs. SF. Dietary intervention could exert a different epigenetic modulation on the immune system in CMA children.

## Introduction

Cow’s milk allergy (CMA) is one of the most frequent food allergies (FAs) in the pediatric age^1^, and is the leading cause of food-induced anaphylaxis in Italian children^2^. CMA prevalence and persistence have been on the rise under the pressure of gene environment interactions leading to immune system dysfunction, mediated at least in part by epigenetic mechanisms^3,4^. Preliminary cross-sectional pilot studies suggest that epigenetic mechanisms drive CMA disease course^5–7^. Immune tolerance is defined as the active suppression of specific immune responses to dietary antigens in the gastrointestinal tract. A subset of regulatory dendritic cells (DCs), expressing CD103, is responsible for delivery of antigen to the draining lymph node and induction of regulatory T cells (Tregs)^8^. Tregs play a pivotal role in immune tolerance^9^. Forkhead box P3 (*FoxP3*) is the major transcription factor that modulates the fate of Tregs^10,11^. The methylation status of FoxP3 is regulated within a highly conserved region within the Treg-specific demethylated region (TSDR), a CpG-rich region^12^. Results from a pilot study showed different *FoxP3* demethylation status comparing CMA children with active disease with those with recent evidence of immune tolerance acquisition^6^. Dietary factors exert a pivotal role in the regulation of epigenetic mechanisms^13^. We observed a significant difference in DNA methylation of T helper (Th)1/Th2 cytokine genes in children who acquired immune tolerance after treatment with extensively hydrolyzed casein formula containing the probiotic Lactobacillus rhamnosus GG (EHCF + LGG) compared to subjects who received other formulas5. Longitudinal studies are needed to elucidate the potential of formula choice in driving epigenetic mechanisms. Current guidelines for the management of CMA suggest that in IgE-mediated CMA infants aged above 6 months, and without a history of anaphylaxis, extensively hydrolyzed formula or soy formula (SF) are appropriate for first line treatment^14^.

The EPICMA (EPIgenetics as target for Cow’s Milk Allergy) trial aimed to compare DNA methylation of *FoxP3*,Th1/Th2 cytokine genes and microRNAs (miRNAs) expression in IgE-mediated CMA children treated with EHCF + LGG vs. SF.

## Results

### Patients and clinical outcomes

From December 2015 to June 2016, 30 consecutive infants with recent evidence (2 to 4 weeks) of signs and symptoms possibly due to IgE-mediated CMA were evaluated. 6 patients were excluded based on the presence of at least one of the exclusion criteria. All patients were receiving standard formula and were weaned. 24 were randomized in the two treatment groups: 12 subjects received EHCF + LGG; and 12 subjects received SF. After 2 to 4 weeks, when a full stable resolution of signs and symptoms occurred, a double blind placebo controlled food challenge (DBPCFC) was performed in all patients. 2 patients from each group were excluded because of a negative DBPCFC. 20 patients (10 per group) resulted positive at the DBPCFC and continued the study. All patients reacted within 2 hours to the first four cow’s milk doses (up to 3 ml of pasteurized cow’s milk) presenting repeated vomiting and nausea, and rash with urticaria.

**Figure 1:**
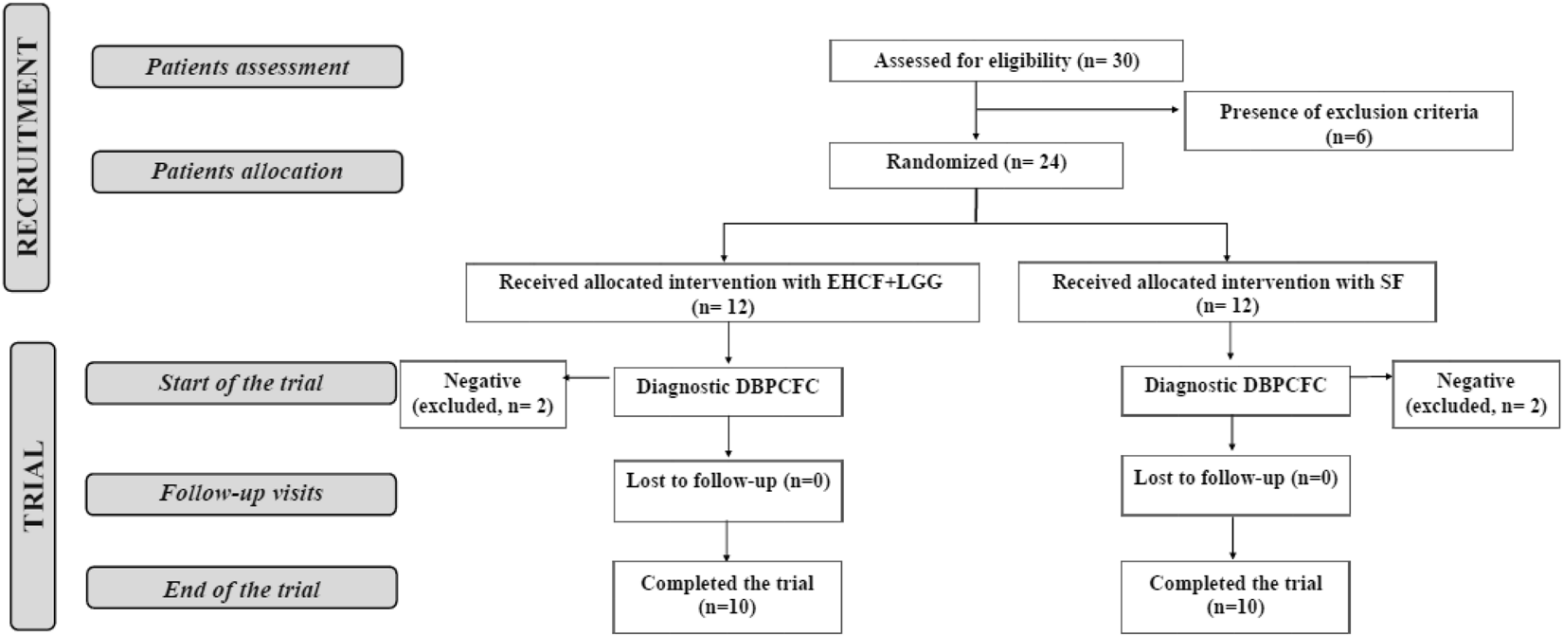
The flow of the patients through the study.

No patient was lost to follow-up (Fig. 1).

Baseline main demographic and clinical characteristics of the study groups are reported in Table 1.

**Table 1:** Baseline features of the subjects enrolled into the study.

	Group 1 (EHCF + LGG)	Group 2 (SF)
N.	10	10
Male, n	5 (50)	550
Cesarean delivery, n (%)	3 (30)	6 (60)
Age at CMA diagnosis (months) (median, IQR)	6.5 (6.0–8.0)	7.0 (6.0–9.0)
Breastfed for ≤ 8 weeks, n (%)	8 (80)	10 (100)
Familial risk of allergy, n (%)	8 (80)	8 (80)
* Father, n (%)*	6 (60)	7 (70)
* Mother, n (%)*	8 (80)	5 (50)
* Sibling, n (%)*	2 (20)	1 (10)
Exposure to passive smoking, n (%)	0 (0)	0 (0)
Positive prick by prick test for fresh milk, (%)	10 (100)	10 (100)
Positive total serum IgE*, n (%)	10 (100)	10 (100)
Positive nBos d 4**, n (%)	10 (100)	10 (100)
Positive nBos d 5**, n (%)	10 (100)	9 (90)
Positive nBos d 6**, n (%)	9 (90)	7 (70)
Positive nBos d 8**, n (%)	10 (100)	10 (100)
Gastrointestinal symptoms at CMA onset, n (%)	6 (60)	5 (50)
*Vomiting, n (%)*	6 (60)	5 (50)
Cutaneous symptoms at CMA onset, n (%)	8 (80)	10 (100)
*Rash and urticaria, n (%)*	8 (80)	10 (100)
Respiratory symptoms at CMA onset, n (%)	4 (40)	4 (40)
*Wheezing, n (%)*	4 (40)	4 (40)

*CMA: cow’s milk allergy; EHCF* + *LGG: extensively hydrolyzed casein formula supplemented with the probiotic LGG; SF: soy formula; IgE: immunoglobulin E; IQR: interquartile range*.

*≥0.35 kU/l.

**≥0.35 kUA/l.

The study groups presented similar demographic and clinical characteristics at the study entry. All subjects were positive for specific IgE and skin prick testing. All patients underwent the planned visits at 6 and 12 months. Adherence to study formula use was optimal, and any deviation was observed. All patients presented a full recovery from CMA signs and symptoms during the follow-up, and body growth pattern was similar in the two study groups. No child was intolerant to the study formulas. No adverse event was attributed to the consumption of the formulas, and no difference was detected in their daily intake.

As reported in Table S1, patients in EHCF + LGG group showed a greater reduction of SPT, IgE, and specific IgE values after 6 and 12 months of dietary treatment compared to children treated with SF. After 12 months of dietary therapy, all patients were subjected to DBPCFC to explore immune tolerance acquisition. 6 of 10 subjects fed with EHCF + LGG acquired immune tolerance, whereas only 2 of 10 subjects treated with SF presented a negative oral food challenge.

### FoxP3 demethylation and expression

**Figure 2:**
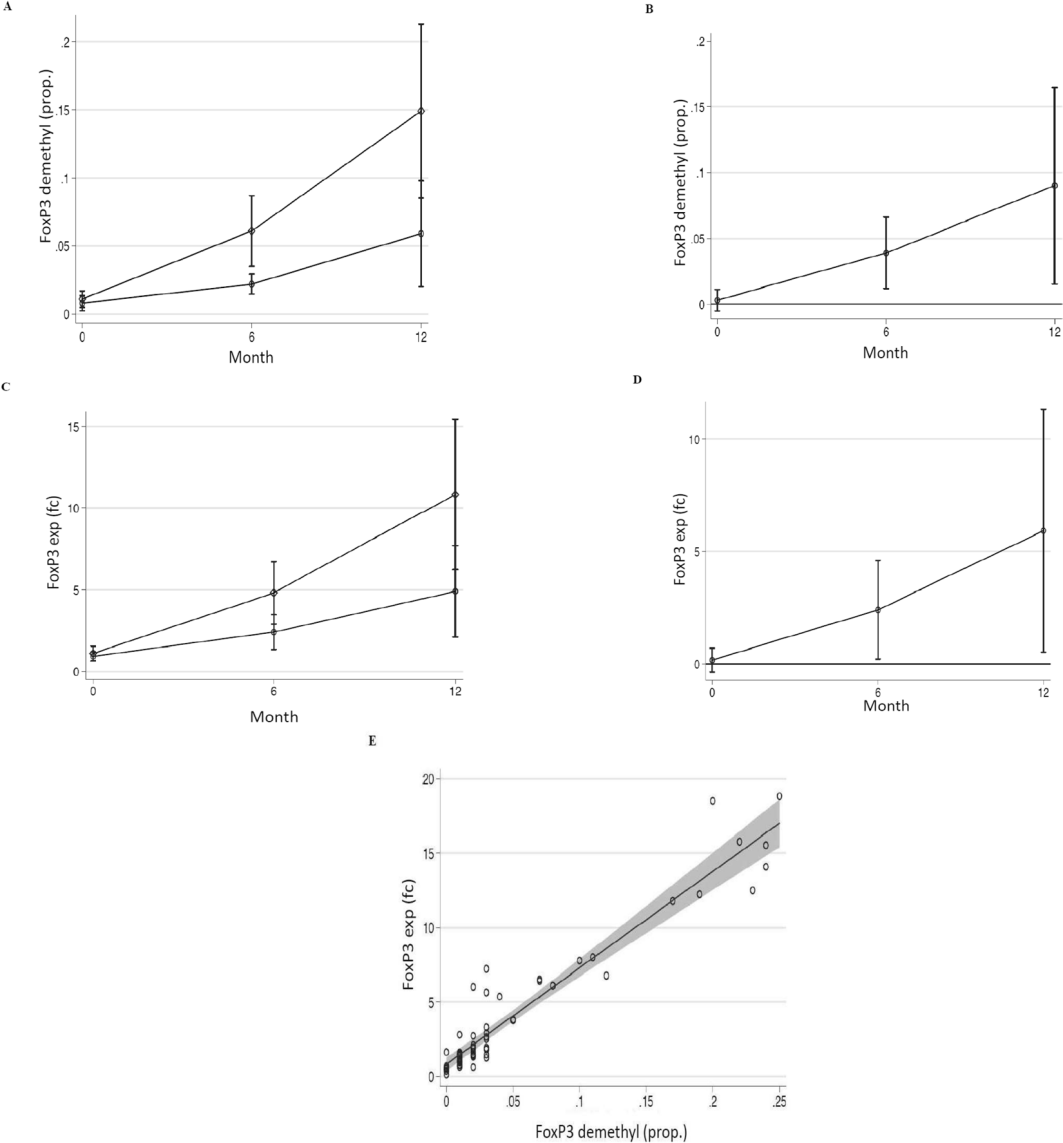
*FoxP3* DNA demethylation and expression. (**A**) *FoxP3* Treg-specific demethylated region (TSDR) demethylation proportion in children enrolled in the EHCF + LGG group (*square*) vs. soy group (*circle*). (**B**) *FoxP3* TSDR demethylation proportion resulted significantly different comparing the two groups at 6 and at 12 months. EHCF + LGG group showed a higher *FoxP3* demethylation proportion compared to SF group (*p* < 0.05). (**C**) *FoxP3 *expression in children enrolled in the EHCF + LGG group (*square*) vs. soy group (*circle*). (**D**) *FoxP3* expression resulted significantly different comparing the two groups at 6 and at 12 months (*p* < 0.05). (**E**) Significant association was observed between *FoxP3* expression and *FoxP3* demethylation proportion in all study subjects at all study points (*p* < 0.01). Plotted values are means and 95% cluster confidence intervals estimated from generalized linear models for fractional or continuous outcomes (see statistical analysis for details). Statistical significance at a *p*-value < 0.05 is present when the 95% confidence interval of the difference does not cross 0.

As shown in Fig. 2A, at the baseline, the *FoxP3* demethylation was similar in the two study groups. Already at 6 months, EHCF + LGG group showed a significant increase of *FoxP3* demethylation rate compared to SF group (Fig. 2B). The difference in *FoxP3* demethylation rates between the two groups further increased at 12 months of dietary treatment (Fig. 2B). *FoxP3* expression levels paralleled its methylation status (Fig. 2C and D). A significant positive association was found between *FoxP3* demethylation rate and respective mRNA expression levels (Fig. 2E).

### Effect of potential confounders on FoxP3 demethylation

We tested whether each discrete baseline confounder (sex, age, mode of delivery, breastfeeding, familial allergy) was associated to *FoxP3* methylation independently of treatment by adding it to the fractional generalized linear models (GLM) as covariable. The between-group change in *FoxP3* methylation was not influenced by any of the confounders (data not shown).

### Th1/Th2 cytokines DNA methylation, mRNA expression and serum profiles

**Figure 3:**
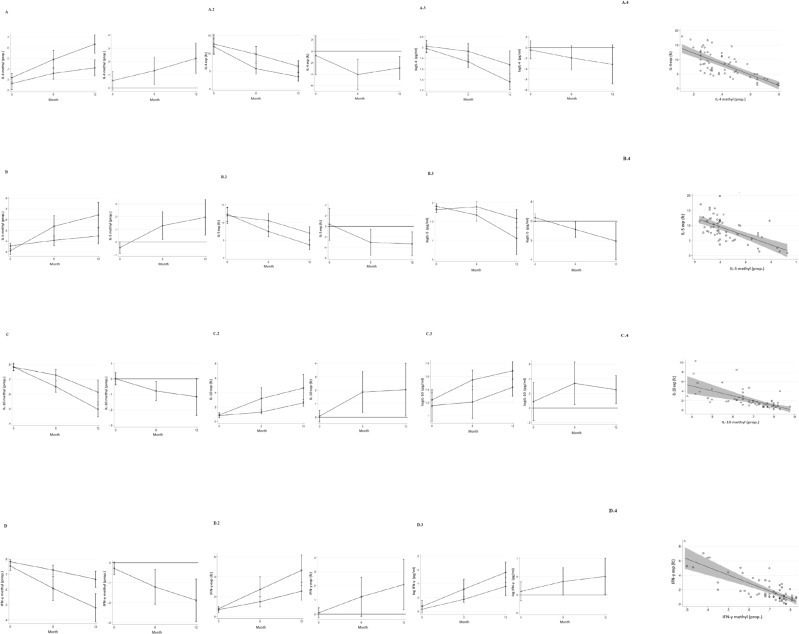
IL-4, IL-5, IL-10, and IFN-γ DNA methylation, expression, and serum levels Time-related changes in IL-4 (**A)**, IL-5 (**B**) and IL-10 (**C)**, IFN-γ **(D**) genes methylation proportion, their expression and serum levels in the EHCF + LGG group (*square*) vs. soy group (*circle*). The statistical differences between the two groups at 6 and 12 months are represented at right side of each panel (*p* < 0.05). There was an association between expression and methylation proportion in all study subjects and time points (*p* < 0.01). Plotted values are means and 95% cluster confidence intervals estimated from generalized linear models for fractional or continuous outcomes (see statistical analysis for details). Statistical significance at a *p*-value < 0.05 is present when the 95% confidence interval of the difference does not cross 0.

Figure 3 shows methylation rate, mRNA expression, and serum levels of IL-4, IL-5, IL-10, and INF-γ. At the baseline, DNA methylation rate, mRNA expression, and Th1/Th2 cytokines serum levels were similar in the two study groups. After 6 months, patients treated with EHCF + LGG presented a higher DNA methylation rate of *IL-4* and *IL-5*. Also, at 6 months, a significant higher reduction of DNA methylation rate of *IL-10* and *IFN-γ* was observed in children treated with EHCF + LGG compared to SF group. Instead, children treated with EHCF + LGG showed lower IL-4 and IL-5, and higher IL-10 and INF-γ mRNA expression and serum levels compared to SF group. These effects were further magnified after 12 months of treatment. Methylation rate of all cytokines was significantly negatively associated with the respective mRNA expression levels (Fig. 3).

### miRNAs expression levels

**Figure 4:**
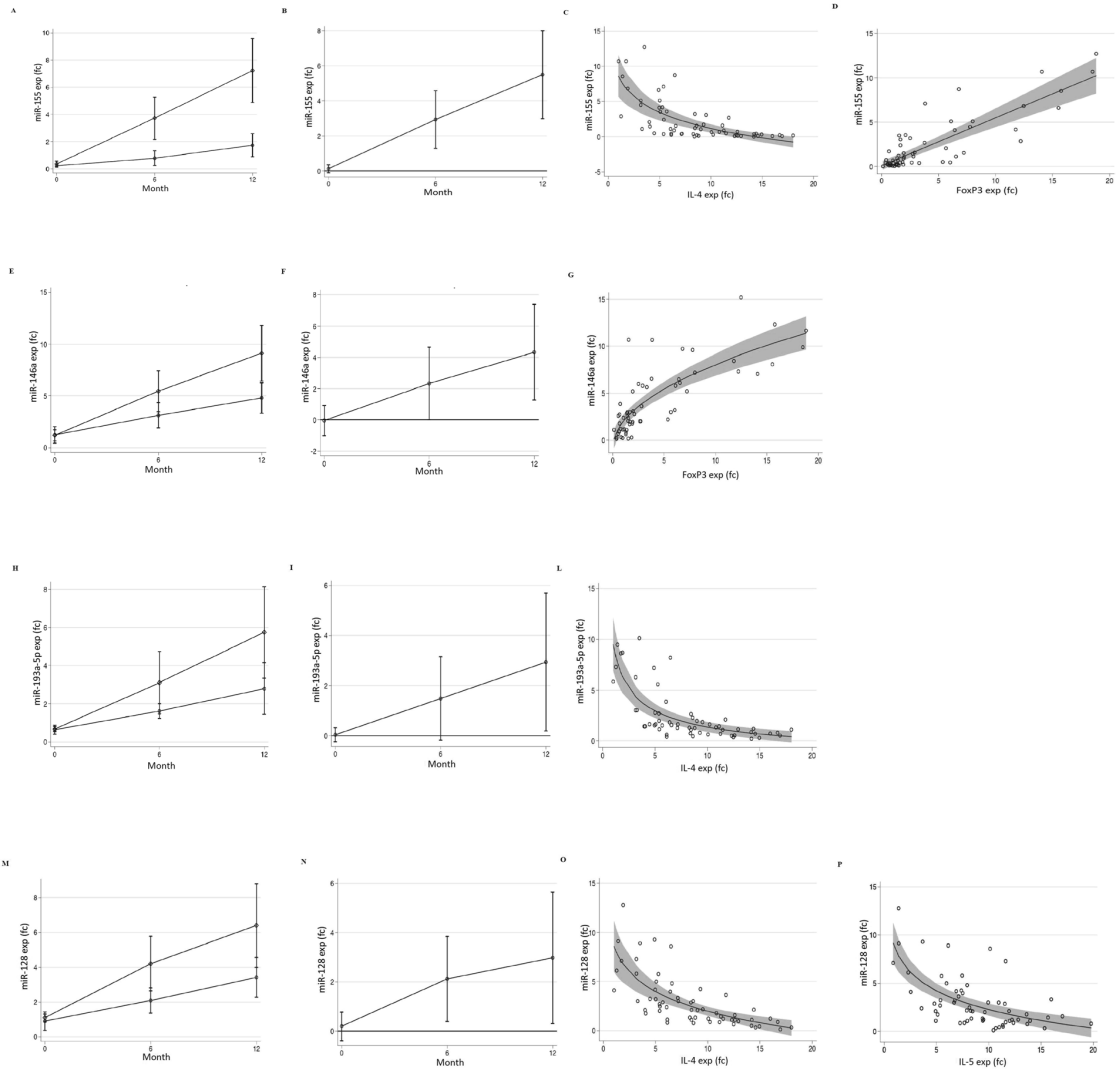
miRNAs expression and their correlation with Th2 cytokines and FoxP3 expression (**A**) Time- related changes in miR-155 expression in the EHCF + LGG group (*square*) vs. soy group (*circle*). (**B**) Significant difference in miR-155 expression was observed at 6 and 12 months comparing the two study groups (*p* < 0.05). A significant association was found with IL-4 (**C**) and *FoxP3* **(D**) expression in all study subjects and time points (*p* < 0.01). (**E**) Time- related changes in miR-146a expression in the EHCF + LGG group (*square*) vs. soy group*(circle)*. (**F**) Significant difference in miR-146a expression was observed at 12 months comparing the two study groups (*p* < 0.05). (**G**) Significant association with *FoxP3* expression was found in all study subjects and time points (*p* < 0.01). (**I**) Time-related changes in miR-193a5p expression in the EHCF + LGG group (*square*) vs. soy group (*circle*). (**H**) Significant difference in miR-193a5p expression was observed comparing the two study groups at 12 months (*p* < 0.05). (**L**) Significant association with and IL-4 expression was found in all study subjects and time points (*p* < 0.01). (**M**) Time-related changes in miR-128 expression in the EHCF + LGG group (*square*) vs. soy group (*circle*). (**N**) Significant difference in miR-128 expression was observed comparing the two study groups at 6 and 12 months (*p* < 0.05). A significant association with IL-4 (**O**) and IL-5 (**P**) expression was found in all study subjects and time points (*p* < 0.01). Plotted values are means and 95% cluster confidence intervals estimated from generalized linear models for fractional or continuous outcomes (see statistical analysis for details). Statistical significance at a *p*-value < 0.05 is present when the 95% confidence interval of the difference does not cross 0.

As shown in Fig. 4, at baseline no significant difference in miR-155, -146a, -128 and -193a expression was observed in the two study groups. After 6 months, an increase of miR-155, -146a, -128, and -193a expression was observed in children receiving both dietary treatments. The miR-155 and miR-128 expression increase was significantly higher in the EHCF + LGG group. After 12 months, miR-155, -146a, -128 and -193a expression levels were significantly higher in the EHCF + LGG group compared to the SF group. MiR-155, -146a, -128, and -193a expression was significantly associated with *IL-4*, *IL-5*, and *FoxP3* expression levels (Fig. 4). No changes in miR-21, -27a, -29a, -126, -145, and -106a expression were observed in the two groups during all study phases (data not shown).

### Exploratory analysis of the association between epigenetics and immune tolerance acquisition

For explorative purposes, we studied the between-group (EHCF + LGG vs. SF), within-time (0, 6 and 12 months) differences in the fractional and continuous outcomes of interest in the subjects in whom CMA tolerance developed *vs*. those in whom it did not develop at 12 months (Table S2). A different pattern of all epigenetic variables (methylation rates and miRNAs expression) was observed in patients in whom immune tolerance developed.

## Discussion

Formula choice for the treatment of IgE-mediated CMA impacts the *FoxP3* methylation status. Our study compared DNA methylation of *FoxP3*, Th1/Th2 cytokine genes and miRNAs expression in IgE-mediated CMA children treated with EHCF + LGG *vs*. SF. We found that the use of EHCF + LGG is associated with a faster and higher *FoxP3* demethylation leading to an up-regulation of its expression. This effect paralleled a higher methylation status of *IL-4*, *IL-5*, a lower methylation status of *IL-10*, *IFN-γ* and selected miRNAs expression toward a Th1 oriented response. *FoxP3* demethylation in Tregs has been associated with immune tolerance induction in peanut allergy^15^. Syed *et al*. demonstrated that subjects who acquired immune tolerance to peanuts had higher numbers of Tregs with higher levels of *FoxP3*demethylation^16^. CMA associated with methylation alterations in Th1/Th2 balance has been described in an epigenome-wide association study^17^. An increase of DNA methylation and consequent decrease of *IFN-γ* expression has been associated with allergy^18^. We found that the use of EHCF + LGG was associated with a significant higher expression of miR-15, -146a, -128 and 193a-5p compared to SF. Overexpression of miR-128 induces an increase of Th1 cell number^19^. A significant decrease in miR-146a expression in children with allergic rhinitis and its positive correlation with *FoxP3* expression has been reported^20,21^. The increase of miR-155 expression in activated T cells leads to the differentiation of Th1 response^22^ and regulates Tregs^23^. A study demonstrated that LGG induced a significant upregulation of miR-155 in human dendritic cells^24^.We demonstrated that miR-193a-5p modulates *IL-4* expression in children with IgE-mediated CMA^7^. We found that EHCF + LGG is able to shape gut microbiota composition increasing butyrate-producing genera^25^. Butyrate as inhibitor of histone deacetylases influences recruitment of DNA methyltransferases to the genes, thus influencing the DNA methylation status^26^. Several studies supported direct HDACs inhibition by butyrate for Tregs induction through an increase of *FoxP3* gene expression^27–30^. In addition, a possible direct effect induced by CpG DNA sequence of LGG on IL-4 and IL-10 expression has been also demonstrated in the FA animal model^31^. Evidence suggest a potential role for casein hydrolysis-derived peptides as modulators of the immune system^32,33^. Our study has several limitations and strengths. We did not provide evidence on which components of the study formulas could be responsible for the modulatory action on epigenetic mechanisms. In addition, despite the number of subjects enrolled was equal to that programmed by the sample size calculation, future studies with a larger sample size and longer duration of follow up are advocated to confirm our results. The strengths of our study are related to the prospective design, to the homogeneous and well-characterized population of patients with definitive CMA diagnosis, and to the number of variables considered.

## Conclusion

A stronger modulation of epigenetic mechanisms associated with a trend toward higher rate of immune tolerance acquisition in children treated with EHCF + LGG has been observed in this study. These results bolster previous findings where a positive effect on immune tolerance acquisition and on the prevention of other atopic manifestations were observed in patients with IgE-mediated CMA treated with EHCF + LGG for a period of 36 months^2,34,35^. The novel data reported herein may serve as a basis for the development of new diagnostic and therapeutic tools for CMA based on epigenetic analysis.

## Methods

### Study subjects

**Figure 5:**
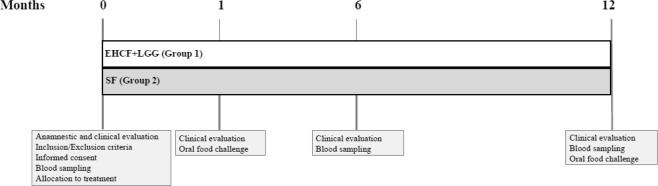
The design of the study.

Children who were consecutively referred at our tertiary center for pediatric allergy because of the recent occurrence of signs and symptoms possibly related to IgE-mediated CMA were considered for the study. The inclusion criteria were age between 6 and 12 months and suspected IgE-mediated CMA (positive clinical history for signs and symptoms possibly related to CMA). The exclusion criteria were: history of cow’s milk protein–induced anaphylaxis; evidence of non-IgE-mediated CMA; concomitant presence of other FAs or other allergic diseases, eosinophilic disorders of the gastrointestinal tract, chronic systemic diseases, congenital cardiac defects, active tuberculosis, autoimmune diseases, immunodeficiency, chronic inflammatory bowel diseases, celiac disease, cystic fibrosis, genetic-metabolic diseases, malignancy, chronic pulmonary diseases, malformations of the gastrointestinal and/or respiratory tract, and administration of prebiotics or probiotics during the 8 weeks before enrollment. Only subjects who met these criteria were invited to participate in the study. Written informed consent was obtained from the parents/tutors of each subject. The study design is depicted in Fig. 5. At the enrollment, full anamnestic and clinical evaluation, skin prick testing, and peripheral venous blood sampling were performed, and according to a randomization list, the patients were randomly allocated to one of two groups of dietary intervention. The first group received EHCF + LGG (Nutramigen, Mead Johnson Nutrition, Evansville, IN, USA); and the second group received SF (Similac Soy Isomil 2, Abbott srl, Rome, Italy). Both study products were commercially available as formula powder for CMA dietary treatment in Italy. Parents received written instruction about the study formula brand name, preparation, and use. The composition of the two study formulas is detailed in Table S3. After 2 to 4 weeks of exclusion diet, when full and stable remission of symptoms was achieved, a DBPCFC was performed as previously described to confirm the diagnosis of CMA^2^. The recruitment continued until a pre-specified number of 10 subjects per group with DBPCFC-proven IgE-mediated CMA diagnosis was achieved. Only subjects with positive DBPCFC continued the study using the formula prescribed at randomization. After 6 months from enrollment subsequent full clinical evaluation, skin prick testing, and blood sampling were performed. Again, the procedures were repeated at 12 months from the enrollment. At that study point a new DBPCFC was performed to explore the possible acquisition of immune tolerance. Unscheduled visits were planned when necessary. Parents or caregivers were asked to keep a daily record of formula use. The amount prepared (millimeters of water and number of formula spoons) and amount left after each consumption were recorded in a diary to assess the amount of formula consumed by the child. Formula use was evaluated at each time visit by dietitians counseling parents about issues that could arise during the elimination diet and on how to reach the daily recommended intake^36^. This allowed the study staff to evaluate compliance with the assigned formula and to ensure that the patients received an appropriate quantity of formula to meet their nutritional requirements. Anamnestic, demographic, anthropometric, and clinical data were obtained from the parents of each patient and recorded in a clinical database together with all the results collected during the study.

### Ethics

The study conducted in accordance with the Declaration of Helsinki, and was approved by the Ethics Committee of the University of Naples “Federico II”, and was registered in the Clinical Trials Protocol Registration System on May 27, 2015 (https://clinicaltrials.gov -ID number: NCT02466035). All methods were performed in accordance with the relevant guidelines and regulations.

### Calculation of sample size

Sample size was calculated taking into account the effect size estimated from a previous study^6^. To detect a difference in 20% in the *FoxP3* demethylation rate in EHCF + LGG group vs. SF group with a power of 80% and a significance level (alpha) of 0.050 using a two-sided two-sample equal-variance t-test we calculated that 10 patients with IgE-mediated CMA per group were needed. However, because the children had to have a DBPCFC-confirmed IgE-mediated CMA diagnosis, we increased the pool of children allocated to the two treatments, and we designed the study to continue patients’ enrollment until at least 10 children per group completed the trial.

### Randomization

Randomization was based on a computer-generated list with consecutive numbers with an allocation ratio of 1:1 between EHCF + LGG and SF groups. Each treatment was numbered according to the randomization scheme without any reference to the group assignment, prepared by a biostatistician not involved in the analysis of study results.

### Skin prick testing

Skin prick tests were performed using fresh whole milk. Reactions were recorded on the basis of the wheal and flare at 15 minutes and considered “positive” if the largest wheal diameter was 3 mm or larger without a reaction to the negative control, as previously described^2^.

### Blood sampling

Twelve mL of peripheral venous blood were collected from each study subject at enrollment, and after 6 and 12 months. An aliquot of 4 mL was collected in a serum separator tube and used for the measurement of total IgE, specific IgE against cow’s milk proteins, and Th1/Th2 cytokines. Samples were centrifuged for 10 min at 3000 rpm. An aliquot of 8 mL was used for epigenetics analysis. Venous blood samples were collected to isolate peripheral blood mononuclear cells (PBMCs), using the Ficoll-Paque (Sigma-Aldrich, St. Louis, MO, USA) method, as described previously^5^. CD4^+^ T-cells were obtained by negative selection using the CD4^+^ T-Cell Isolation Kit II (Miltenyi Biotec, Bergisch Gladbach, Germany). Non-target cells were labeled with a cocktail of biotin-conjugated monoclonal antibodies (MicroBead Cocktail, Miltenyic Biotec) and the magnetically labeled non target T cells were retaining on a column in the magnetic field of a separator (Miltenyi Biotec). This protocol produces >95% pure CD4^+^ T cells, as tested by fluorescence-activated cell sorting analysis. CD4^+^ T cells obtained were processed for DNA and RNA extraction.

### Total IgE, specific IgE and Th1/Th2 cytokines serum levels

Total IgE and specific IgE against of cow’s milk proteins (nBos d4; nBos d5; nBos d6; nBos d8) were assessed by enzymatic immunoassay (Phadia 100 ThermoFisher Scientific CAP system, Rodano Milano, Italy). Measurements were expressed as kilounits per liter (kU/L).

The concentrations of IL-4 and IL-10 were measured with a Human IL4/IL10 Enzyme immunoassay kit (Boster Biological Technology, Ltd., Fremont, CA, USA). The IL-5 and IFN- concentrations were measured using the human ELISA assay kit (BioVendor, Brno, Czech Republic). The minimum detection concentrations were 15.6 pg/mL for IL-4, 7.8 pg/mL for IL-5 and IL-10, and 0.78 pg/mL for IFN-γ.

### Methylation-sensitive high-resolution melting (MS-HRM) and sequencing

DNA was extracted from CD4^+^ T-cells using the DNA Extraction Kit (GE Healthcare, Little Chalfont, UK). Methylation studies were performed by methylation-sensitive high-resolution melting (MS-HRM), as previously described^37^. One µg of extracted DNA was modified with sodium bisulfite using the EZ DNA Methylation Gold Kit (ZYMO Research Co., Orange, CA, USA), according to the manufacturer’s instructions. The converted DNA was stored at −20 °C until used. The primers used for DNA methylation analysis of IL-4, IL-5, IL-10, IFN-γ, and *FoxP3* was designed *in silico*, using MethPrimer (http://www.urogene.org/methprimer/) and are reported elsewhere^5,6^. Real-time PCR was performed with the LightCycler® 480 instrument (Roche Applied Science, Penzberg, Germany) using 96-well plates (Roche Applied Science). Extensive optimization experiments were performed in order to maximize PCR amplification efficiency, including PCR program parameters, Mg^2+^, primer and template concentrations. Sodium bisulfite-converted DNA (15 ng) was added to the PCR reaction mix, which consisted of high-resolution melting Master Mix (Roche Applied Science), 0.25 μM primers, and Mg^2+^ (2.5 mM). dH_2_O was used to supplement up to 20 μl. The real-time PCR protocol began with one cycle at 95 °C for 10 min followed by 40 cycles of 95 °C for 10 s, 61 °C for 10 s, and 72 °C for 10 s. Immediately after amplification, a re-annealing cycle consisting of 95 °C for 1 min and a rapid cooling to 65 °C for 1 min was introduced to prepare the melting curve acquisition step. Real-time fluorescence acquisition was set at the elongation step (72 °C). Samples whose amplification begun late and sample whose relative fluorescence value on the raw melting-curve plot was low were not further processed. All PCR reactions were performed in triplicate for each sample. Melting data acquisition began at 69 °C and ended in 95 °C, using a ramp rate of 0.2 °C/s. High-resolution melting analysis was also performed with the LightCycler® 480 instrument (Roche Applied Science) using 96-well plates (Roche Applied Science). Data processing included normalization and resulted on the normalized melting curves and the respective negative derivative of fluorescence over the temperature plots, using the LightCycler 480® gene scanning software. The settings for data collection were 50 fluorescence acquisition points per degree centigrade resulting on a ramp rate of 0.01 °C/s. Comparison of the melting curve or the peaks of an unknown sample with those of the controls gave the semi-quantitative estimation for the methylation level of that sample. The results were confirmed by direct sequencing (Sanger method modified: dideoxy-nucleotide-tri phosphates [ddNTPs] labeled with four different fluorophores) and analyzed by capillary electrophoresis (analytical specificity and sensitivity of the test: >99%).

### Th2 and Th1 cytokines, FoxP3, and selected miRNAs expression analysis

RNA was extracted from the CD4^+^ T cells using the Trizol protocol (Invitrogen, Life Technologies Europe BV, Monza, Italy), and quantified with the NanoDrop 2000c spectrophotometer (Thermo Scientific, Waltham, MA, USA), as previously described^7^. For complementary DNA (cDNA) synthesis, 1 μg total RNA was transcribed with a High Capacity cDNA Reverse Transcription kit (Applied Biosystems, Foster City, CA, USA) according to the manufacturer’s instructions. Quantitative real-time PCR (qRT-PCR) analysis of *IL-4*, *IL-5*, *IL-10*, *IFN-γ*, and *FoxP3* was performed with the TaqMan gene expression assay kit (Applied Biosystems, Grand Island, NY, USA) according to the manufacturer’s instructions. Samples were run in triplicate at 95 °C for 15 s and 60 °C for 1 min using an ABI Prism 7900 HT (Applied Biosystems). The quantitative gene expression was calculated with the comparative Ct method and normalized against the Ct of glyceraldehyde 3-phosphate dehydrogenase (*GADPH*) messenger as reference gene. For miRNAs expression analysis, we selected miRNAs that have been reported^38^ to play pivotal roles in T cell function: hsa-miR-21-5p, -27a-5p, -29a-5p -128-1-5p, -146a-5p, -126-5p, -155-5p, -145-5p,-106a-5p, and -193a-5p. Five μg/μL of the RNA templates was used for cDNA synthesis with the Universal cDNA synthesis kit (Exiqon, Vedbaek, Denmark). cDNA samples were evaluated for miRNA expression in 7900 HT (Applied Biosystems, Foster City, CA, USA). Specific primers were chosen and provided from the universal miCURY LNA primer set (Exiqon, Vedbaek, Denmark). Universal miCURY LNA 5S rRNA and U6 snRNA were used as reference genes for relative quantification. Samples were run in triplicate at 95 °C for 10 s and 60 °C for 1 min with a ramp rate of 1.6 °C/s for melting curve analysis, using an ABI Prism 7900 HT (Applied Biosystems). Data analysis was performed with the comparative threshold cycle (CT) method.

### Statistical analysis

Most continuous variables had non gaussian distribution and all are reported as median and interquartile range. The concentration of IL-4, IL-5, IL-10, and IFN-γ was log10-transformed to reduce skewness and to meet the assumptions made by the GLM described in the next paragraphs. The between-group (EHCF + LGG vs. SF) change in the fractional outcomes of interest (*FoxP3*, *IL-4*, *IL-5*, *IL-10* and *IFN-γ* methylation) was studied using a fractional GLM with a logit link. Such GLM employed time (discrete: 0 = 0; 1 = 6 months; 2 = 12 months), treatment (discrete: 0 = SF; 1 = EHCF + LGG), and a time × treatment (discrete × discrete) interaction as predictors and used cluster confidence intervals to take into account the repeated measures^39,40^. The between-group (EHCF + LGG vs. SF) changes in the continuous outcomes of interest (*FoxP3* expression, IL-4 expression, IL-4 concentration, IL-5 expression, IL-5 concentration, IL-10 expression, IL-10 concentration, IFN-γ expression, IFN-γ concentration, miR-155, miR-146a, miR-193a5p and miR-128 expression) were studied using a GLM with a gaussian family and an identity link. Such GLM employed time (discrete: 0 = 0; 1 = 6 months; 2 = 12 months), treatment (discrete: 0 = SF; 1 = EHCF + LGG) and a time × treatment (discrete × discrete) interaction as predictors and used cluster confidence intervals to take into account the repeated measures^41^. For the GLM using *FoxP3* demethylation as outcome, we tested the potential confounding effect of sex (discrete: female = 0; male = 1), age (discrete: 0 = < 6 months; 1 = ≥ 6 months), delivery (discrete: 0 = normal; 1 = cesarean), breastfeeding (discrete: 0 = no; 1 = yes), and familial allergy (discrete: 0 = no; 1 = yes) by comparing the between-group (EHCF + LGG vs. SF) time-averaged difference stimated by the GLM described previously to that estimated by a GLM adding the potential confounder as co-variable^33^. The relationship between the expression of a given outcome vs. its methylation or concentration or the methylation and concentration of another outcome was modeled in study subjects at all time points using a GLM with a gaussian family and an identity link and cluster confidence intervals to take into account the repeated measures^41^. We used degree 1 multivariable fractional polynomials to test whether the outcome-predictor relationships were linear^42^. A log-transformation of the predictor was used to linearize the following relationships: miR-155 expression vs. IL-4 expression, miR-128 expression vs. IL-4 expression, and miR-128 expression vs. IL-5 expression; a square root transformation of the predictor was used to linearize the following relationship: miR-146a expression vs. *FoxP3* expression; an inverse square root transformation of the predictor was used to linearize the following relationship: miR- 193a5p expression vs. IL-4 expression. For explorative purposes, we used the GLMs described aboveto study the between-group (EHCF + LGG vs. SF), within-time (0, 6, and 12 months) differences in the fractional and continuous outcomes of interest in the subjects in whom immune tolerance developed vs. those in whom immune tolerance did not develop at 12 months. Such GLMs were identical to those previously described except that they employed time (discrete: 0 = 0; 1 = 6 months; 2 = 12 months), tolerance at 12 months (discrete: 0 = no; 1 = yes), and a time × tolerance (discrete × discrete) interaction as predictors^41^. Statistical analysis was performed using Stata 15.1 (Stata Corporation, College Station, TX, USA).

## Supplementary information

Supplementary Table
