# Publisher Correction: Randomized controlled trial on the influence of dietary intervention on epigenetic mechanisms in children with cow’s milk allergy: the EPICMA study

**DOI:** 10.1038/s41598-019-45226-8

**Published:** 2019-06-26

**Authors:** Lorella Paparo, Rita Nocerino, Cristina Bruno, Carmen Di Scala, Linda Cosenza, Giorgio Bedogni, Margherita Di Costanzo, Maurizio Mennini, Valeria D’Argenio, Francesco Salvatore, Roberto Berni Canani

**Affiliations:** 1 Department of Translational Medical Science, University Federico II, Naples, Italy; 2 Clinical Epidemiology Unit, Liver Research Center, Basovizza, Trieste, Italy; 3 Hospital Bambino Gesù in Rome, Vatican City, Italy; 4 CEINGE-Biotecnologie Avanzate s.c.ar.l., University Federico II, Naples, Italy; 5 Department of Molecular Medicine and Medical Biotechnologies, University Federico II, Naples, Italy; 6 European Laboratory for the Investigation of Food-Induced Diseases, University Federico II, Naples, Italy; 7 Task Force on Microbiome Studies, University Federico II, Naples, Italy

Correction to: *Scientific Reports* 10.1038/s41598-019-38738-w, published online 26 February 2019

A supplementary file containing Tables S1, S2 and S3 was omitted from the original version of this Article due to a technical error. This has been corrected in the HTML and PDF versions of the Article.

