# Supplementary material for: Randomized controlled trial on the influence of dietary intervention on epigenetic mechanisms in children with cow’s milk allergy: the EPICMA study
Source: Sci Rep. 2019 Feb 26;9:2828. doi: 10.1038/s41598-019-38738-w (PMC6391485; doi:10.1038/s41598-019-38738-w)
Supplement: Supplementary file 1 — Supplementary Table [file 41598_2019_38738_MOESM1_ESM.pdf]

Randomized controlled trial on the influence of dietary intervention on epigenetic mechanisms in children with cow's milk allergy: the EPICMA study

Lorella Paparo[1,4†], paparolorella@gmail.com

Rita Nocerino[1,4†], ritanocerino@alice.it

Cristina Bruno[1], cristinabruno88@libero.it

Carmen Di Scala[1], carmendiscala@gmail.com

Linda Cosenza[1], lindacosenza@libero.it

Giorgio Bedogni[2], giorgiobedogni@gmail.com

Margherita Di Costanzo[1], mara.dicostanzo@live.it

Maurizio Mennini[3], maurizio.mennini@gmail.com

Valeria D'Argenio[4,5,7], dargenio@ceinge.unina.it

Francesco Salvatore[4,5,7], salvator@unina.it

Roberto Berni Canani[1,4,6,7], berni@unina.it

[†]Equal contributors

[1] Department of Translational Medical Science, University Federico II, Naples,, Italy

[2]Clinical Epidemiology Unit, Liver Research Center, Basovizza, Trieste, Italy

[3]Division of Allergy, Bambino Gesù Children's Hospital, Vatican City, Vatican City

[4]ImmunoNutritionLab at CEINGE-Biotecnologie Avanzate s.c.ar.l. Naples, Italy, University Federico II, Naples, Italy

[5]Department of Molecular Medicine and Medical Biotechnologies, University Federico II, Naples, Italy

[6]European Laboratory for the Investigation of Food-Induced Diseases, University Federico II, Naples, Italy

[7]Task Force on Microbiome Studies, University Federico II, Naples, Italy

Supplementary Table 1. Skin prick test, total and specific serum IgE values during the study.

	Group 1 (EHCF + LGG)			Group 2 (SF)		
	**T0**	**T6**	**T12**	**T0**	**T6**	**T12**
**Skin prick test to whole milk (mm)**	0.76 (0.51 to 1.02)	0.51** (0.36 to 0.67)	0.06*** (0.01 to 0.12	0.99 (0.77 to 1.21)	0.96 (0.81 to 1.11)	0.93 (0.80 to 1.05)
**Total IgE (kU/l)**	359 (273 to 445)	324 (228 to 421)	195 * (114 to 276)	437 (297 to 576)	391 (254 to 527)	386 (257 to 516)
**nBosd 4 (kUA/l)**	10.2 (4.6 to 15.8)	5.8 (1.5 to 10.2)	2.6 (0.36 to 4.7)	16.7 (3.4 to 30.0)	19.7 (2.01 to 37.4)	16.5 (-1.65 to -34.6)
**nBosd 5 (kUA/l)**	8.4 (4.8 to 11.9)	3.4 (1.0 to 5.9)	1.7 (0.4 to 3.0)	9.0 (3.5 to 14.6)	7.7 (3.9 to 11.5)	4.6 (2.05 to 7.07)
**nBosd 6 (kUA/l)**	4.58 (1.41 to 7.76)	2.12 (0.15 to 4.07)	1.17 (0.21 to 2.56)	3.01 (0.86 to 5.16)	2.47 (0.55 to 4.4)	0.87 (0.11 to 1.63)
**nBosd 8 (kUA/l)**	33.0 (12.5 to 53.6)	16.6 (-0.2 to 33.3)	13.1 (-3.2 to 29.4)	28.9 (10.0 to 47.9)	25.0 (5.7 to 44.0)	17.9 (-0.4 to -36.2)

**p<0.05, **p<0.01 and *** p<0.001*

Values are means and cluster 95% confidence obtained from generalized linear models for continuous outcomes (see statistical analysis for details).

Supplementary Table 2. Effects of epigenetic mechanisms on immune tolerance acquisition

	Group 1 (EHCF + LGG)			Group 2 (SF)		
	T0	T6	T12	T0	T6	T12
**Fox P3 demethylation rate (prop)**	0.013 (0.007 to 0.020)	0.08 *** (0.05 to 0.99)	0.21 *** (0.20 to 0.24)	0.007 (0.001 to 0.011)	0.018 (0.014 to 0.022)	0.028 (0.022 to 0.34)
**IL to 4 methylation rate (prop)**	0.29 (0.23 to 0.37)	0.51 (0.40 to 0.61)	0.65 ** (0.54 to 0.76)	0.28 (0.24 to 0.32)	0.37 (0.32 to 0.42)	0.44 (0.37 to 0.51)
**IL to 5 methylation rate (prop)**	0.35 (0.32 to 0.38)	0.60 *** (0.51 to 0.69)	0.73 *** (0.62 to 0.84)	0.32 (0.29 to 0.36)	0.39 (0.35 to 0.42)	0.42 (0.39 to 0.45)
**IL to 10 methylation rate (prop)**	0.77 (0.75 to 0.81)	0.64 * (0.60 to 0.69)	0.47 ** (0.40 to 0.53)	0.78 (0.77 to 0.80)	0.72 (0.69 to 0.75)	0.61 (0.55 to 0.67)
**INF γ methylation rate (prop)**	0.75 (0.72 to 0.79)	0.57 ** (0.49 to 0.65)	0.43 *** (0.34 to 0.51)	0.80 (0.76 to 0.79)	0.73 (0.71 to 0.76)	0.67 (0.63 to 0.71)
**Fox P3 expression (fc)**	1.43 * (1.08 to 1.78)	6.02 *** (4.66 to 7.38)	14.9 *** (12.9 to 16.9)	0.76 (0.49 to 1.02)	2.02 (1.12 to 2.93)	3.19 (1.99 to 4.39)
**Log_{10}IL to 4 concentration (pg/ml)**	2.00 (1.89 to 2.1)	1.72 (1.6 to 1.9)	1.30 (1.1 to 1.5)	2.00 (1.92 to 2.1)	1.90 (1.78 to 2.02)	1.66 (1.48 to 1.84)
**Log_{10} IL to 5 concentration (pg/ml)**	1.85 (1.77 to 1.90)	1.68 (1.50 to 1.85)	0.80 ** (0.39 to 1.14)	1.87 (1.79 to 1.94)	1.85 (1.72 to 1.99)	1.68 (1.59 to 1.78)
**Log_{10} IL to 10 concentration (pg/ml)**	1.33 (1.18 to 1.50)	1.58 (1.38 to 1.78)	1.80 *** (1.70 to 1.90)	1.11 (0.93 to 1.30)	1.24 (1.04 to 1.44)	1.4 (1.32 to 1.48)
**Log_{10} INF γ concentration (pg/ml)**	0.20 (0.04 to 0.44)	1.04 ** (0.80 to 1.30)	1.72 *** (1.55 to 1.89)	0.08 (-0.06 to 0.22)	0.34 (0.22 to 0.45)	0.78 (0.54 to 1.01)
**miR193 a5p (fc)**	0.84 (0.60 to 1.10)	3.86 * (2.20 to 5.50)	7.87 *** (6.65 to 9.09)	0.55 (0.43 to 0.68)	1.37 (1.19 to 1.54)	1.88 (1.58 to 2.17)
**miR155 (fc)**	0.39 (0.19 to 0.59)	4.05 * (2.20 to 5.90)	7.89 ** (5.3 to 10.4)	0.24 (0.11 to 0.37)	1.05 (0.45 to 1.65)	2.20 (1.04 to 3.39)
**miR146 a (fc)**	1.20 (0.59 to 1.81)	6.89 ** (5.4 to 8.37)	10.0 ** (7.89 to 12.1)	1.21 (0.52 to 1.91)	2.56 (1.80 to 3.31)	4.95 (3.10 to 6.82)
**miR128 (fc)**	1.03 (0.76 to 1.30)	4.76 * (3.10 to 6.40)	7.89 ** (5.89 to 9.90)	0.98 (0.53 to 1.43)	2.07 (1.42 to 2.72)	2.91 (2.18 to 3.64)

** p<0.05, ** p<0.01 and *** p<0.001*

Values are point estimates and cluster 95% confidence obtained from generalized linear models for fractional or continuous outcomes(see statistical analysis for details).Prop=proportion; fc=fold increase.

Supplementary Table 3. Composition of the study formulas (x 100 g).

Nutrient	Units	Extensively hydrolyzed casein formula with LGG (EHCF+LGG)	Soy Formula (SF)
Energy	kcal	500	459.2
Protein	g	14	11.25
Carbohydrate	g	55	47.7
Fat	g	25	25
Linoleic acid	g	4.5	4.6
α-linolenic acid	mg	340	-
Arachidonic acid	mg	170	-
Docosahexaenoic acid	mg	85	-
Vitamin A	μg	450	413.10
Vitamin D	μg	7.5	6.90
Vitamin E	mg	6.7	4.70
Vitamin C	mg	105	2.07
Thiamin (B1)	mg	0.4	0.28
Riboflavin (B2)	mg	0.45	0.43
Niacin	mg	5	6.4
Vitamin B6	mg	0.3	0.28
Folic Acid	μg	80	73.6
Vitamin B12	μg	1.5	2.15
Biotin	μg	15	21.5
Vitamin K	μg	65	50.7
Panthothenic Acid	mg	2.5	3.5
Choline	mg	120	110
Inositol	mg	85	23
Calcium	mg	565	505
Sodium	mg	240	211.4
Phosphorus	mg	390	362.8
Iron	mg	9	8.7
Magnesium	mg	50	36.4
Zinc	mg	3.5	3.6
Potassium	mg	610	505
Chloride	mg	480	298.5
Manganese	mg	0.3	0.11
Copper	mg	0.38	0.36
Iodine	μg	105	73.6
Selenium	μg	11	9.3
Chromium	μg	11	-
Molybdenum	μg	28	-
Lactobacillus rhamnosus GG	CFU	$1x10^8$	-
